# Motion Analysis in Alpine Skiing: Sensor Placement and Orientation-Invariant Sensing

**DOI:** 10.3390/s25082582

**Published:** 2025-04-19

**Authors:** Behrooz Azadi, Michael Haslgrübler, Alois Ferscha

**Affiliations:** 1 Pro2Future GmbH, Altenberger Strasse 69, 4040 Linz, Austria; michael.haslgruebler@pro2future.at; 2 Institute of Pervasive Computing, Johannes Kepler University, Altenberger Straße 69, 4040 Linz, Austria; ferscha@pervasive.jku.at

**Keywords:** inertial measurement unit, motion analysis, sensor fusion, sensor placement and orientation, alpine skiing monitoring

## Abstract

In alpine skiing, accurate and real-time estimation of body pose and inclinations due to turning is critical as it demonstrates the skier’s turning behavior and abilities. Although inertial measurement units (IMUs) ease measuring kinematics in extreme conditions and provide such indications of skiers’ behavior, they often suffer from sensor placement and orientation variability. This study explains the impact of sensor placement and orientation on the captured signals and proposes a preprocessing algorithm that can rotate raw signals from various locations and orientations similar to those near the Center of Mass (CoM). The preprocessing algorithm involves a sensor fusion approach using a quaternion-based complementary filter (CF) to rotate raw signals and extract turning motions via the global wavelet spectrum. Our experiment, validated on data collected from 14 sensors including two smartphones placed on different body parts during skiing sessions, demonstrates that the preprocessing algorithm can effectively reconstruct side motions, represent skiing turns, and detect turns independent of sensor placement and orientation. In field experiments with six skiers, the suggested preprocessing algorithm consistently detected skiing turns with an overall RMSE of 0.77 and MAE of 0.50 on all of the sensors relative to a reference sensor.

## 1. Introduction

Alpine skiing is a highly dynamic winter sport that requires skiers to have precise coordination of the lower body, core, and upper body to maintain balance and control speed while performing skiing turns safely and smoothly [1]. A motion capture system to monitor skiers’ behavior while executing turns will be advantageous in providing evidence-based feedback on their performance, especially when capable of long-term monitoring. Long-term monitoring might be particularly beneficial to gain a deeper understanding of performance slumps [2] and injury mechanisms [3] as it provides information in a prolonged period and potentially addresses the data scarcity [4]. In recent years, alpine skiing motion tracking and analysis using wearable [5,6] and visual [7,8] sensors has significantly progressed. Perhaps the inertial measurement unit (IMU) is the most preferred wearable sensor in many sports motion analyses, particularly individual sports including alpine skiing [9], since it is easy to attach and does not suffer from environmental issues such as occlusion. In the alpine skiing context, scholars have employed IMU sensors to detect turns [10], recognize skiers’ activities [11], and evaluate their performance [12,13,14,15].

Alpine skiing turns are complex actions that involve regular changes in direction. The ski turn is an essential technique for regulating speed in skiing competitions, where the goal is to make turns with minimal speed loss. The outcome is influenced by various factors such as frequency, total duration, radius, and speed of movement [16]. Recreational ski turns differ from ski racer turns in terms of speed, tempo, and rhythm [17]. In recreational skiing, the skier determines all elements together with the line of descent. In ski racing, these elements are determined by the gates set up on the slope. Regardless of the subject group, it is necessary to examine skiing turns as the basic units of alpine skiing activities to assess alpine skiers’ performance and abilities. Therefore, an essential feature of an alpine skiing monitoring system is turn detection to extract turns for further analysis.

Yu et al. [18] coined the term turn detection and compared the detected number of turns via IMU sensors against the ground truth. They extracted the zero crossing of the roll angle of an IMU sensor located on the pelvis and defined the time interval between two consecutive zero crossings as a turn. Similarly, Martinez in [10] experimented with gyroscope measurements in the lab setting and proved the method in the field to detect turns. Martinez et al. [19] reviewed turn detection methods and demonstrated the possibility of accurately detecting turn switches with different sensors, depending on the specific needs, such as sensor placement or equipment available, e.g., type of IMU. In [20], the authors used the boot roll angle to visualize a turn cycle and illustrated the roll picks as the middle of the turn and zero crossing as the point between two turns. Therefore, the number of picks in the signal represents the number of performed turns. Similarly, [21] used a zero crossing of the Center of Mass (CoM) Y-axis to detect the turn switch. However, the highlighted methods are limited to the sensor location and orientation and are validated by expert skiers. Additionally, there are varied turn detection methods, where scholars employed a visual sensor [22], pressure insole [23], or more than one sensor [24], which are not considered in this study; a comparison, however, is provided by [19].

Although investigations on skiing monitoring, turn detection, and performance analysis have achieved valuable results, scholars have always used fixed sensor placement and orientation [5,14,25,26,27], and most studies have attached several sensors [20,21,28] in different locations on the skier’s body. Consequently, the authors developed preprocessing algorithms based on the sensor setup assumptions. Although full body sensing is suitable and accurate for analyzing data to know the skiing mechanism, it does not necessarily apply to all skiing scenarios. Developing a motion capture system is even more crucial when targeting recreational skiers since it needs scalability and considering variabilities in the real-world environment. In [29,30], the authors argue that orientation change can cause a distribution shift and, consequently, accuracy drops in human activity recognition. Also, Zhong et al. [31] pointed out the dependency of gait analysis models on the sensor placement. In real life, recreational alpine skiers cannot always promise a fixed sensor location and orientation during a prolonged skiing day, which may hurt data analyses such as turning style recognition and turn detection. Additionally, employing several sensors might not be suitable for long-term data recording, as it is not scalable and flexible. Moreover, it has not yet been experimented on how the aforementioned analyses/algorithms are affected by the sensor location and orientation. Also, the algorithm may fail in the case of sensor misplacement, considering full body sensor setup can be overwhelmingly complex and error-prone.

In contrast to the other approaches, this study aims to develop an alpine skiing sensing approach to ease the continuous monitoring of skiers’ activities and behaviors without requiring an overwhelming sensor setup, i.e., either many sensors or specific and inflexible sensor setups. We experiment with replacing several sensors with only one IMU sensor embedded in smartphones and implementing a preprocessing algorithm so that the subject is not concerned about the sensor’s location and orientation. However, it can add several orientation and location changes during the data recordings. The preprocessing algorithm must be able to transform signals from an arbitrary location and orientation to the Optimal Reference Frame and therefore correct all the orientation changes during the recording. Thus, a skier will have the freedom of choice and flexibility to attach their sensor without being concerned about an exact sensor location and orientation. For the large population of recreational alpine skiers, such monitoring enables skiers to record their activities seamlessly using their smartphones and receive information regarding their activities and performance.

### Problem Statement

The outcome of studies on turn detection suggests that the best place to observe skiing turns is near the Center of Mass (CoM). Let us assume we attach an imaginary sensor to the CoM in a way that the X-axis points up against the gravity vector and is perpendicular to the transverse plane, the Z-axis points forward in the direction of movement and is perpendicular to the coronal plane, and the Y-axis points to the side and is perpendicular to the sagittal plane, according to the anatomical plane. Accordingly, we can see turning behavior on the Y-axis of the accelerometer. Let us call this the Optimal Reference Frame to describe skiing turns. Problem articulation: If a sensor attachment deviates from the optimal attachment, due to misplacement or misorientation, or if it decides to locate the sensor in an arbitrary place on the body, how can we recover the turning behavior? Through this investigation, we will endeavor to find answers to the following questions:

- How do sensor location and orientation affect the proposed monitoring algorithms in other studies, especially turn detection?
- Is there any sensor setup to collect the most informative data independent of sensor location and orientation?
- Can the recorded signals from a sensor positioned on the body, with an arbitrary location and orientation, be rotated to the Optimal Reference Frame?

Finding an answer to these questions is crucial because it allows for flexibility in data collection. Hypothesis: We hypothesize that via a sensor fusion algorithm, which combines the accelerometer, gyroscope, and magnetometer, we can transform raw data into the Optimal Reference Frame to see the turning behavior on a specific axis. We offer a preprocessing algorithm for motion analysis, which eases turn detection and addresses potential data collection issues via smartphones, namely sensor location and orientation change. We adapt a standard sensor fusion technique, a quaternion-based complementary filter to combine the accelerometer, gyroscope, and magnetometer, to a challenging, real-world problem—dealing with arbitrary sensor placements during skiing. Furthermore, we detect the body inclinations due to turning behavior using a wavelet analysis.

## 2. Materials and Methods

To test our hypothesis and answer the proposed question, we first introduce the Skier Fixed Reference Frame to address potential technical issues of data collection using smartphones’ IMU.

### Data Collection

**Figure 1:**
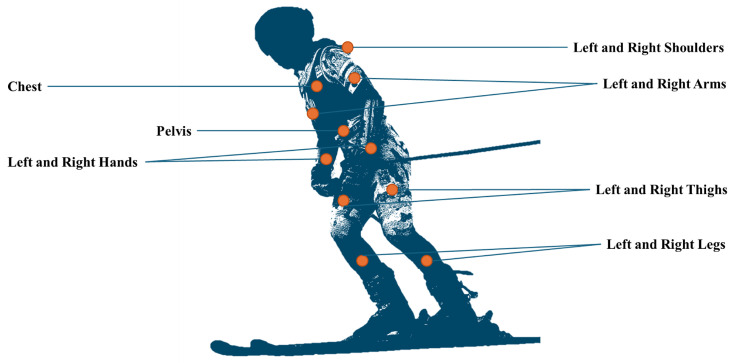
Attached Xsens sensors on the skier’s body.

Two data collection sessions were arranged to record skiing activities using Xsens sensors (Xsens MTw Awinda) and smartphone IMU (Samsung Galaxy S9). The Xsens sensors were attached to the shoulders, arms, hands, thighs, and legs (all left and right). Two Xsens sensors were also located on the chest and pelvis. Moreover, two smartphones were placed on the left and right side of the pelvis, see Figure 1. The smartphone’s IMU returns acceleration and angular velocity signals with a frequency of 500 Hz and the Earth’s magnetic field at 100 Hz frequency. The smartphone Samsung Galaxy S9 (model SM-G960F) contains an LSM6DSL IMU (an acceleration range of ±2/±4/±8/±16 g and an angular rate range of ±125/±245/±500/±1000/±2000 dps) [32] and an AK09916C compass (a sensing range of 4.7~5.2 mT) [33]. We used the Physics Toolbox Sensor Suite, version 2025.01.29, to access these built-in sensors. Xsens MTw Awinda Wireless Motion Tracker can support the simultaneous connection of up to 20 sensors and time-synchronized data sampling. The sampling rate was set to 60 Hz and the data were recorded by at least 50 Hz [34].

The full body sensor setup allows us to examine the effect of sensor location and orientation on skiing patterns and experiment with the existing approaches in turn detection. One can assume sensor locations simulate the location change, and sensors attached to the hands naturally simulate the orientation change during data recordings. Moreover, we will investigate sensor fusion to rotate raw signals and examine if it can transform signals from an arbitrary frame to an Optimal Reference Frame, where turning behavior can be seen on a particular axis.

Data collection took place in varied locations and seasons, in November at Dachstein and July at Hintertux, with different conditions in variables such as snow quality and slopes. Consequently, we may observe additional noise levels that contributed to the recorded signals due to the ski piste conditions. Six skiers with different mastery levels participated in these two data recordings. Skiers performed various skiing techniques chosen by an experienced alpine skiing instructor based on the Austrian Ski Instruction Teaching Plan [35], including parallel ski steering—long radii, parallel ski steering—short radii, dynamic parallel ski steering—long radii, dynamic parallel ski steering—short radii, carving—long radii, and carving—long radii. Having recorded various turning styles lets us examine skiing motions with different dynamics, considering the variability they add to the data regarding turn radius and rapid change in direction or body pose. Table 1 summarizes the data acquisition process. In the first session at Hintertux, subjects performed twelve runs, and in the second session at Dachstein, participants performed ten skiing runs.

The data were gathered with at least a 50 Hz sampling rate, and all the recorded signals were resampled to 50 Hz, which contains sufficient information for high-frequency activities [36]. We filmed skiing sessions using a GoPro camera for data labeling and further analysis. The collected data were visualized and presented to the experienced alpine skiing instructor technique by technique. We plotted recorded signals from the pelvis and chest as the reference sensors. Based on the finding of [18] and data visualizations, it was confirmed that we can rely on these two sensors as the ground truth.

## 3. Skier Fixed Reference Frame

What complicates sports analysis is that the sensor frame is not necessarily aligned with the body frame. While in devices or airplanes the sensor orientation is identical to the object orientation, this does not hold the same for humans. Humans tend to be flexible in locating their sensors, especially when utilizing smartphones, or move their body parts, which causes an orientation change. Consequently, body movement cannot be seen on the same axis of an IMU in the case of orientation change.

**Figure 2:**
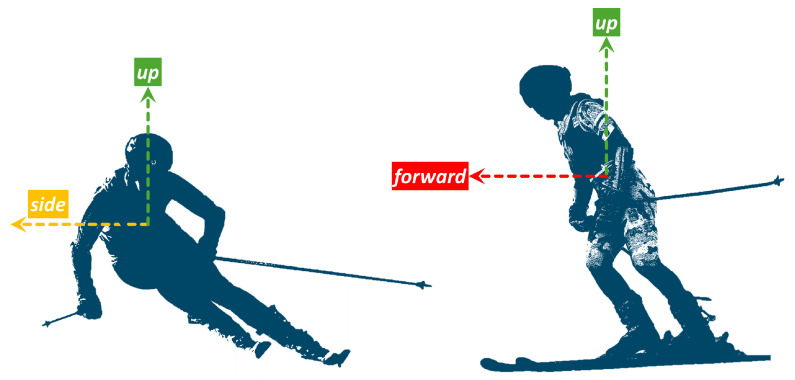
Skier Fixed Reference Frame. The reference frame front view and side view depicts different vectors of the SFRF.

To track skiers’ motions, we introduce the Skier Fixed Reference Frame (SFRF), Figure 2, which has three axes: up, forward, and side. In the SFRF, the up vector is aligned but reversed to the gravity vector and points up, the forward vector is aligned with the direction of movement, and the side vector is pointing from the CoM to the side of the subject. Thinking of the anatomical plane coordinate systems, the side, forward, and up coordinates align with the right, anterior, and superior, respectively. This reference system is aligned with the orientation of an imaginary sensor attached to the CoM. As already discussed in the literature [18], having a sensor located on the pelvis near the CoM allows us to detect and analyze skiing turns.

Such a reference frame helps us to analyze turning behavior by looking at side motions as acceleration due to turning, and body inclination to the left and right are represented on the side motion. It is worth noting that movements around the forward direction affect the side direction. Moreover, forward motion represents the acceleration due to leaning forward and backward. Similarly, the acceleration due to jumping or bending is observable on the up vector.

## 4. Preprocessing Algorithm

To transform recorded signals from the sensor frame to the SFRF, we developed a two-step sensor preprocessing algorithm consisting of sensor fusion and side motion detection. First, we combine the accelerometer, gyroscope, and magnetometer from each sensor, following the quaternion-based orientation tracking introduced in [37] and Chapters 9.1 and 9.2 of [38]. Quaternions do not suffer from singularities in contrast with Euler angles [39]. This method calculates a rotation quaternion at each time step to rotate acceleration signals. The filter updates the rotation quaternion with every new recording to provide instantaneous attitude estimations. As a result, gravity will be projected on the up vector (Y-axis) and the forward and side motions on the other axes (X- and Z-axes). In the second step, we find the side motions among axes on the horizontal plane (X- and Z-axes) by applying a wavelet transform on the skiing activities.

### 4.1. Sensor Fusion

For each sample, we calculate a rotation quaternion to transfer the acceleration values to fix the tilt and project the acceleration due to gravity on one of the axes. To do so, we need a rotation quaternion $q_{rot}=q_{w}+iq_{x}+jq_{y}+kq_{z}$, which can be computed using a rotation of θ radian around an axis **v**:

(1) $$q\left(\theta ,v\right)=cos\left(\frac{\theta }{2}\right)+iv_{x}sin\left(\frac{\theta }{2}\right)+jv_{y}sin\left(\frac{\theta }{2}\right)+kv_{z}sin\left(\frac{\theta }{2}\right)$$

Let $\omega =(\omega_{x},\omega_{y},\omega_{z})$ be the current angular velocity reading. Using the current gyroscope measurement, we can calculate a rotation quaternion $q_{\Delta }$, which represents the instantaneous rotation from the sensor frame:

(2) $$q_{\Delta }=q(\Delta t||\omega ||,\frac{\omega }{\left||\omega |\right|})$$

Considering $q^{\left(t\right)}$ as the accumulated quaternion rotations, we can compute the current rotation quaternion as

(3) $$q_{\omega }^{\left(t+\Delta t\right)}=q^{\left(t\right)}*q_{\Delta }$$

Using the quaternion estimation from gyroscope readings, we can rotate the accelerometer readings to have a fixed up vector align with the gravity vector:

(4) $$q_{acc\_up}=q_{\omega }^{\left(t+\Delta t\right)}*q_{acc}^{\left(raw\right)}*q_{\omega }^{{\left(t+\Delta t\right)}^{-1}}$$

where $q_{acc}^{\left(raw\right)}=0+ia_{x}+ja_{y}+ka_{z}$ is a vector quaternion of the raw accelerations in the sensor frame, $a=(a_{x},a_{y},a_{z})$.

Due to the drift in the gyroscope measurements, added noise, and inaccurate starting orientation, there can be a small angle between the computed up vector and gravity (the real up vector). Using the normalized vector component of $q_{acc\_up}$, we can calculate the rotation angle and the rotation axis. Note that the normalized vector is perpendicular to the following:

(5) $$v=\frac{\left({q_{acc\_up}}_{x},{q_{acc\_up}}_{y},{q_{acc\_up}}_{z}\right)}{\left|\right|q_{acc\_up}\left|\right|}$$

(6) $$n=\left(v_{x},v_{y},v_{z}\right)\times \left(0,1,0\right)=\left(-v_{z},0,v_{x}\right)$$

(7) $$\Phi =cos^{-1}\left(v_{y}\right)$$

Then, we fuse the accelerometer and gyroscope via a complementary filter to fix this error:

(8) $$q_{c}=q\left(\left(1-\alpha_{c}\right)\Phi ,\frac{n}{\left||n|\right|}\right)q_{\omega }^{\left(t+\Delta t\right)}$$

We can replace $q_{\omega }^{\left(t+\Delta t\right)}$ in Equation (4) by the updated rotation quaternion $q_{c}$.

Considering *m* as the current calibrated magnetometer reading and $mag_{ref}$ a measurement at the beginning of recording, we have

(9) $$m_{ref}^{\prime }=q_{ref}*m*q_{ref}^{-1}$$

and

(10) $$m^{\prime }=q*m*q^{-1}$$

where $q_{ref}$ and *q* are the corresponding rotation quaternions. In a perfect world, there is no difference between $m_{ref}^{\prime }$ and $m^{\prime }$. By projecting them on the horizontal plane, we can detect if there is an error using their angular difference:

(11) $$\theta =arctan2(m_{x}^{\prime },m_{z}^{\prime })$$

and

(12) $$\theta_{ref}=arctan2\left(m_{ref,x}^{\prime },m_{ref,z}^{\prime }\right)$$

Finally, using a complementary filter for the second time, we will have

(13) $$q_{rot}=q\left(\left(1-\alpha_{y}\right)\left(\theta -\theta_{ref}\right),\left[0,1,0\right]\right)*q_{c}$$

The $\alpha_{c}$ and $\alpha_{y}$ are complementary filter parameters, and their values should be close to one to reduce the gain as much as possible.

### 4.2. Side Motion Detection

**Figure 3:**
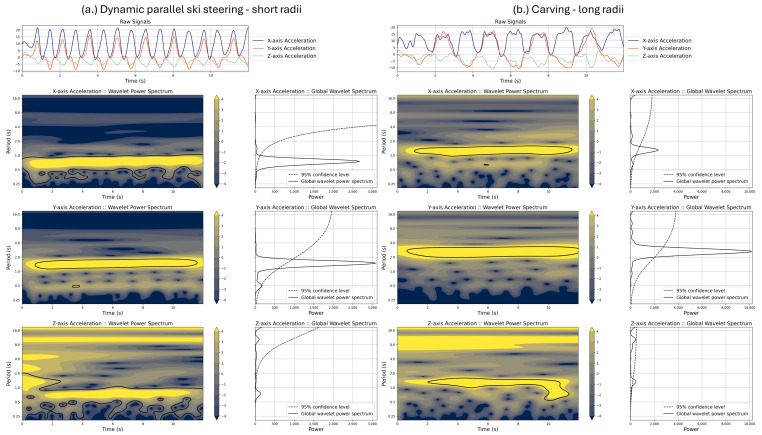
The figure illustrates the wavelet power and global wavelet spectrums for two turning styles: (**a**) Dynamic parallel ski steering - short radii and (**b**) Carving - long radii. The Y-axis of the accelerometer attached to the chest displays clear patterns of the skier’s turning motions on the raw signals, which hold the highest energy in the global wavelet spectrum among all three axes.

Body inclinations due to turning yield periodic patterns on the IMU sensors [10,15,20,40] with a high range of acceleration and orientation. Thus, they might have a higher power when looking at wavelet results [41,42]. Among the recorded signals using the sensor attached to the chest, the Y-axis of the accelerometer demonstrates clear patterns of skiing turns. We also found that the Y-axis of this sensor constantly demonstrates the highest global wavelet spectrum when looking at the wavelet analysis results. Figure 3 illustrates the results of the wavelet analysis, where one can compare three accelerometer axes in the time and frequency domain. Figure 3 displays the global wavelet spectrum for each accelerometer channel, where the Y-axis created the highest peak.

**Figure 4:**
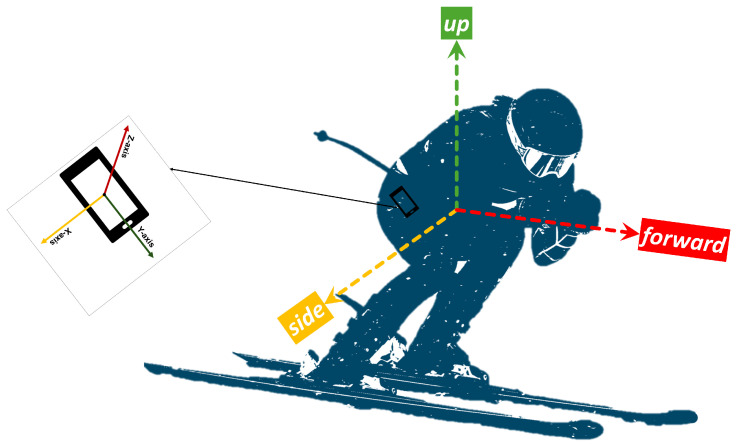
Skier Fixed Reference Frame 3D. The Skier Fixed Reference Frame is defined by three axes—side, forward, and gravity—to make it easier to understand and analyze data. This system also allows skiers to attach their sensors in any position and orientation. The recorded signals, via an IMU, will be transformed from the sensor frame into the Skier Fixed Reference Frame by fusing the accelerometer, gyroscope, and magnetometer, and also using global wavelet spectrum, the side and forward motions will be detected.

Accordingly, we apply a wavelet transform on the X- and Z-axes after the sensor fusion (the Y-axis is already labeled as the up vector) to detect the side motion between these accelerations on the horizontal plane. The motion related to the side motions has a higher power spectrum and creates a higher peak in the global wavelet spectrum. The other signal, with lower power, is regarded as the forward motion. Figure 4 demonstrates the sensor rotation.

## 5. Turn Detection

Assuming the attached sensor near the CoM, e.g., the pelvis or chest, as the ground truth [18,20,21] (X-axis aligned with gravity and Y-axis points to the side), the X-axis of the gyroscope [18], roll angle [20], and Y-axis of the accelerometer [21] in the body frame depicts the turning behavior, where peaks and valleys are the turning points and zero crossing is the turn switch. Similarly, such turning patterns are visible on the side motions in the Skier Fixed Reference Frame regardless of sensor location and orientation. On the other hand, the recorded signals using accelerometers and gyroscopes are varied depending on the sensor location and orientation.

The converted signals are filtered using a second-order low-pass Butterworth filter with a cut-off frequency of 2 Hz to eliminate noise. We applied a strong filter since the noise caused by severe skiing conditions is more than usual compared to other human activities or laboratory settings [43], where the authors examined low-pass filters with cut-off frequencies ranging from 0.5 Hz to 12 Hz. However, in our case, a cut-off frequency of 2 Hz balanced sufficient turn detection with noise reduction. Moreover, smartphones, in the real world, are not fixed on the body and may move in the pocket, which can introduce more noise. Additionally, applying such a strong filter helps to avoid unwanted turn detection events due to creating more peaks. It is worth mentioning that extreme filtering methods can hurt activity patterns and have an adverse effect on other scenarios, e.g., activity recognition. Dissimilar to the turn detection, in the data visualization and other analyses in the study, we applied a cut-off frequency of 4 Hz.

## 6. Results

### 6.1. Transformation

The sensor fusion algorithm first combines raw accelerometer, gyroscope, and magnetometer measurements from each sensor independently to compute rotation quaternions instantaneously. Applying rotation quaternions to the raw 3D acceleration transforms acceleration from the sensor frame to the world reference frame, explained in Section 4.1, which aligns the Y-axis with the direction of gravity. The preprocessing algorithm then applies a wavelet transform on the X- and Z-axes to identify the signal with the highest power, which represents the side direction and is used for turn detection. To compare rotated signals to the raw signals and the ground truth, we segmented signals into 12 s, equal to the shortest activity in the dataset.

**Figure 5:**
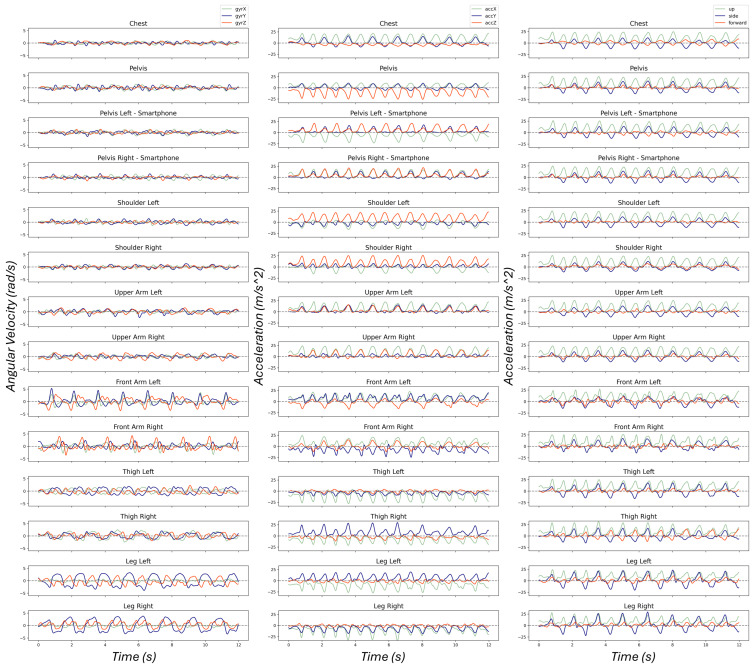
Recorded signals using sensors attached to different locations of the body. **Left column** displays angular velocity in the sensor frame. **Middle column** demonstrates signals recorded by accelerometers in the sensor frame. **Right column** depicts the result of the preprocessing algorithm (sensor fusion and side motion detection) in the Skier Fixed Reference Frame. As one can see, patterns change considerably under the effect of sensor location and orientation (**left and middle columns**), which get even more crucial if turn detection algorithms are developed considering a fixed sensor location and orientation. Moreover, zero-crossing values on a particular axis (applied in the other studies) can vary due to recorded patterns on the accelerometer and gyroscope, comparing collected signals from the chest and pelvis.

The combination of the preprocessing steps should deliver a stable turning pattern regardless of sensor placement and orientation. Let us again consider the sensor attached to the chest as the reference sensor. Then, we can observe skiing patterns and turns on the Y-axis of the accelerometer. However, as Figure 5 exhibits, only the acceleration recorded by the Y-axis of the sensor located on the pelvis displays similar patterns. Moreover, skiing patterns on the accelerometer channels vary when changing the sensor location. The same holds for the gyroscope measurements. Although the angular velocities measured by the X-axis of dissimilar sensors sometimes demonstrate similar patterns, they do not always display the skiing turns clearly. Additionally, the captured patterns using the gyroscope of different sensors are dissimilar, especially the upper arms and front arms.

On the other hand, the outcome of the proposed preprocessing algorithm permanently exhibits similar turning behavior on the side motion in contrast to the raw measurements recorded by the accelerometer and the gyroscope. As a result, independent of the sensor location and orientation, we can capture similar skiing patterns in the Skier Fixed Reference Frame in contrast to the sensor frame. Figure 5 illustrates the impact of sensor placement on the raw measurements in the left and middle columns and the preprocessing algorithm on the right column for dynamic parallel ski steering—short radii recorded in the second data collection session at Dachstein. Although this turning style requires fast movements and rapid body inclinations, some sensors do not represent turning patterns evidently, e.g., gyro readings from sensors attached to arms and accelerometer readings from sensors attached to legs.

**Figure 6:**
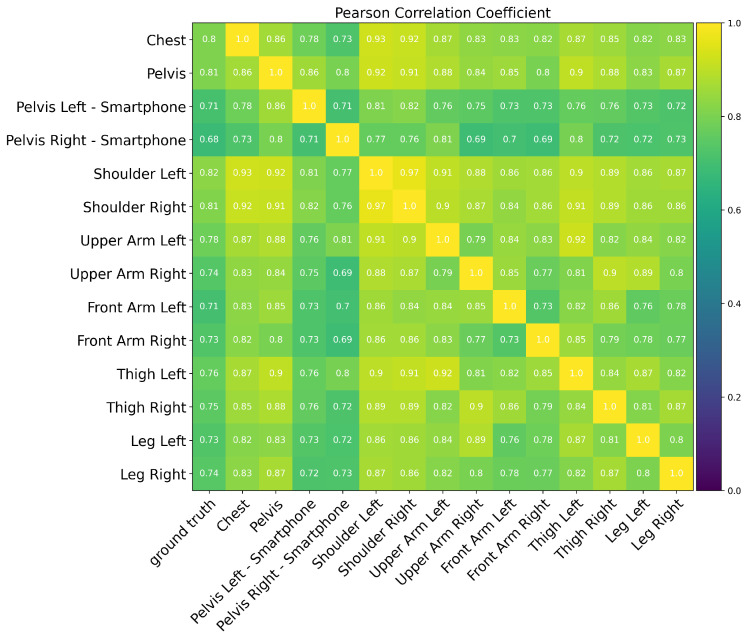
Correlation analysis illustrates a high similarity between the detected side motions and captured turning behaviors on the Y-axis of the ground truth sensor. The lowest correlation to the ground truth belongs to the smartphone attached to the right pelvis.

Moreover, we computed the correlation between the detected side motion and the captured turning behavior on the ground truth sensor using Pearson product-moment correlation coefficients. The correlation analysis result demonstrates a high agreement of side motions extracted from each sensor after the transformation to the ground truth and among all the sensors, Figure 6.

**Figure 7:**
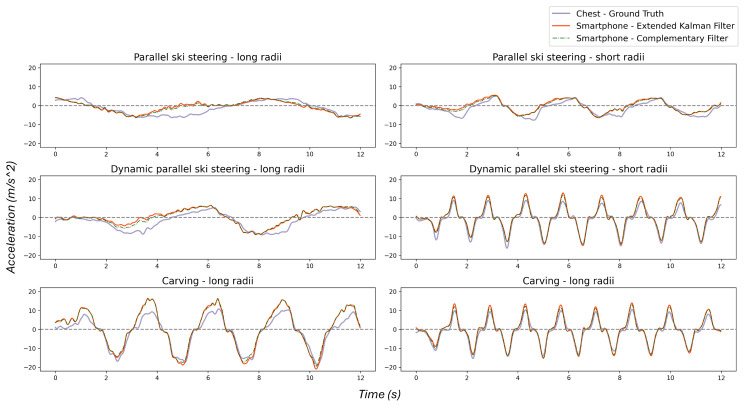
Side motion per technique in comparison with the ground truth, the Y-axis of IMU sensor attached to the chest, and EFK as a benchmark.

Additionally, Figure 7 shows an example of the side motions for all the techniques compared against the ground truth (Y-axis of the Xsens placed on the chest) and the Extended Kalman Filter (EFK) as a benchmark. The side motions are extracted from the smartphone attached to the right pelvis.

### 6.2. Turn Detection

**Figure 8:**
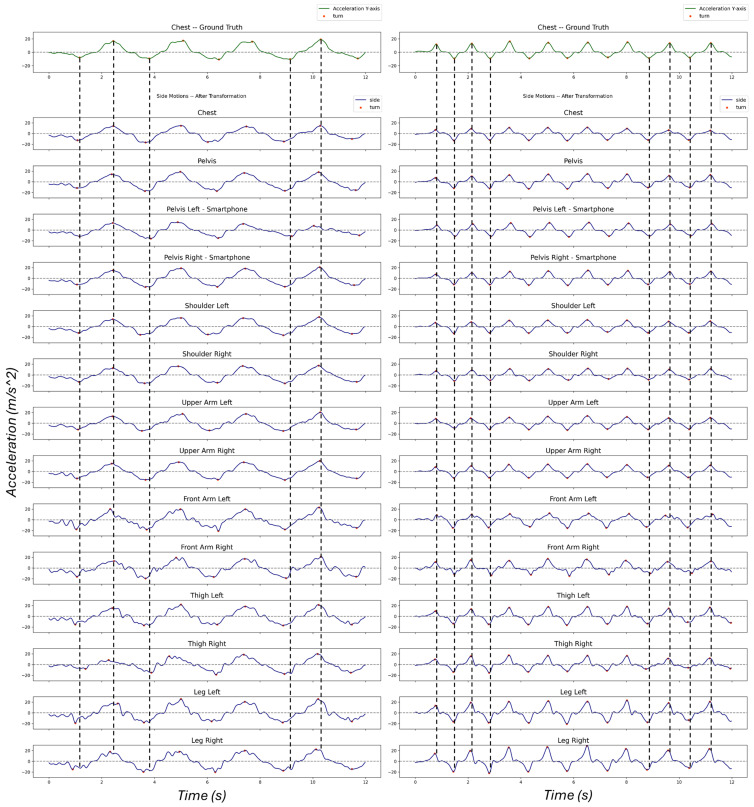
Turn detection comparison with the ground truth. Left column presents a long radius and right column illustrates a short radius style.

Figure 8 illustrates one sample of turn detection, where one can compare the outcome of extracted turns from each sensor using the side motion. Except for some artifacts in the lower body, the other signals show turning points at almost the same time. Regardless of such an error, similar turning behavior is reflected on the side motion everywhere.

No matter which approach from the literature we apply to the raw signals, the results of turn detection will not be stable unless we use a fixed sensor location and orientation. In other words, zero-crossing values and peaks and valleys, applied in the previous studies, can vary due to the sensor position on the skier’s body. As a result of transforming skiing signals to the SFRF, independent of the sensor location and orientation, we can capture turning patterns on the side motions. Table 2 presents turn detection results extracted from the side motion, the roll angle computed by raw accelerations, the X-axis of the gyroscope (gyrX), and the Y-axis of the accelerometer (accY). We compare these values with the ground truth and a baseline (five turns per sample) to report their effectiveness. The metrics are computed based on the measurements coming from all of the sensors. RMSE is helpful when a detection method delivers a value far from the ground truth as it penalizes outliers severely. MAE explains the average absolute difference between the detected and actual number of turns. Assuming the turn detection rate as the number of detected turns divided by the actual number of turns, a correct turn detection must result in one. Therefore, the standard deviation of an accurate turn detection should be close to zero.

**Figure 9:**
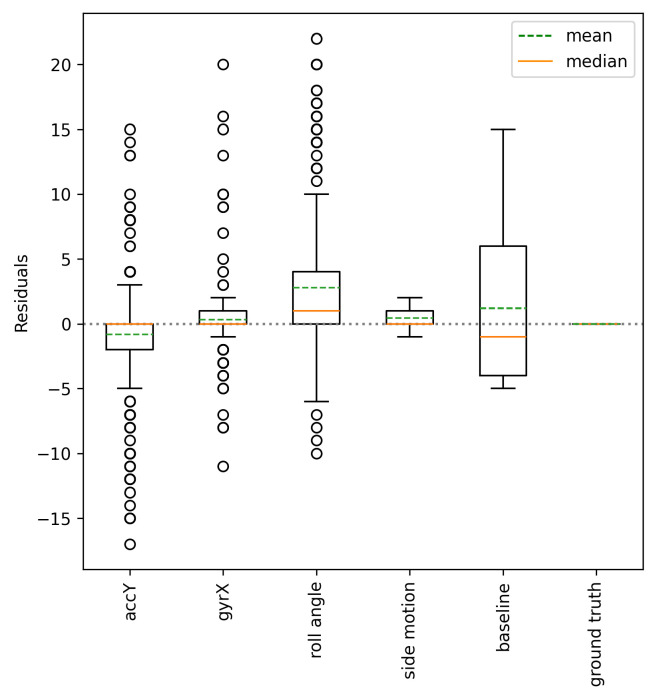
Turn detection residuals. Extracted turns from the side motions exhibit no outliers in contrast to the other signals.

Figure 9 illustrates the box-plot of the residuals using each approach. The results show that the side motion, regardless of the skier, sensor location, and technique, consistently produces a precise number of detected turns with small inter-quartile ranges (IQRs) and a mean close to zero, very similar to the median of residuals. On the other hand, the box-plot for the number of detected turns from other signals displays large inter-quartile ranges (IQRs) (except gyrX), including significant outliers, which indicates inconsistency. The inaccurate turn detection results are due to varied turn patterns captured by dissimilar sensors, as seen in Figure 5.

Table 3 reports the turn detection performance using each sensor and each input signal. As indicators suggest, the Y-axis of the accelerometer (accY) delivers varying values except for the pelvis. The X-axis of the gyroscope (gyrX) shows more reliable outcomes. However, it still looks very faulty, especially for the shoulders. On the other hand, the number of extracted turns from the side motion is very accurate independent of the sensor placement.

## 7. Discussion

This study attempts to explain the impact of sensor location and orientation on alpine skiing motion capture and provides a preprocessing algorithm to alleviate the corresponding issues. The proposed method involves sensor fusion using a quaternion-based complementary filter and side motion detection using the global wavelet spectrum. The complementary filter combines the gyroscope and accelerometer to fix the gravity (tilt correction). It afterward fuses the magnetometer to correct the yaw (rotation around the gravity) and set a reference to one axis (toward the north). The algorithm applies a wavelet transform on the rotated signals to detect the side motions, showing the skier’s turning behavior.

Other filters: The complementary filter in the first step can potentially be replaced by other popular filters, such as Madgwick, Mahony, or EKF, to compute rotation quaternions. Regardless of the filter used for the rotation, it needs extra processing to catch the side motions. Compared with the other filters, the CF is easy to implement and has a low computational complexity [44,45], which makes it a proper candidate for real-time processing on edge devices.

Global reference frame: In motion tracking, we may describe the subject’s motion about a reference frame. There are various coordination systems to be used depending on the case. We choose the global reference frame defined by three axes: east, north, and up (called local tangent plane coordinates (LTPs) or the east north up (ENU) local tangent plane). Using an IMU, we can theoretically estimate the north via magnetometer measurements and the up vector using the gravity recorded by the accelerometer. If a skier drives in line with one of the axes on the horizontal plane (let us assume the north), accelerations due to body inclinations will be seen only on this axis. But in the real world, a skier rarely descends a slope precisely aligned with one of the axes in the horizontal plane. Therefore, we need to adjust the direction of movement to be aligned with one of the horizontal axes. Using physics and biomechanics, we know that if the skier executes turns around the line at 45 degrees, the produced accelerations will be shared equally between the horizontal axis. So, if the rotated signals on the horizontal plane are duplicated, the maximum adjustment is 45 degrees. Also, if signals are dissimilar, the rotation is zero. Knowing this simple fact, we can utilize a similarity metric to find if the signals on the horizontal plane are equally distributed. As a result, the adjustment angle to rotate signals in the horizontal plane is similarity_coefficient∗45 degrees. This idea needs further investigation. We experimented with the cosine similarity metrics, which did not improve the outcome significantly.

Correlation: It is worth noting that the outcome of the preprocessing algorithm does not necessarily match the captured signals from the chest or any other sensor near the CoM since every body part may contribute extra acceleration. The sensors attached to the legs and arms display such an effect adequately as they experience different dynamics than the trunk. Additionally, side motion is an estimation of the body pose by fusing accelerometers, gyroscopes, and magnetometers. Therefore, the quality of sensors will impact the final result, e.g., front arms introduce more noise. In other words, the preprocessing algorithm brings the signals from any arbitrary location to the same coordinate as the CoM. Also, there might be a little bit of shift as the entire body does not tilt at the same time to produce a turn, which is more visible in techniques performed with longer radii or lower speeds, such as parallel steering—long. Moreover, smartphone sensors have a different quality than the Xsens sensors and are manually synchronized with the other Xsens sensors. Therefore, any of these parameters can hurt the correlation result. These may explain discrepancies among sensors in the correlation analysis. However, the smartphone with the worst correlation is depicted in Figure 7.

Euler angles: When using Euler angles, it is necessary to know the sensor orientation. Euler angles are mostly explained in a static sensor orientation [46], while during skiing, the body orientation and, therefore, sensor orientation will be different than the initial orientation, e.g., front arms. Additionally, the dynamics of skiers are too complicated to avoid the gimbal lock, particularly for turns with high speeds and inclinations, such as carving—long radii, where a rotation about 90 degrees around the pitch may not be unusual, e.g., getting in a sitting position. These effects may explain the high error in turn extraction using roll.

Turn detection analysis: For detecting turns, we applied the most basic approach to annotate peaks and valleys as turns. Although this approach is employed frequently and is straightforward, it might not be very accurate, especially for the basic turning styles, where the turn cycle is rather long and body inclination due to turning is low. Furthermore, turns are not just points but rather a very short period, which may vary depending on the underlying turning technique. However, considering the potential errors in turn detection, extracting turns using side motion exhibits a consistent behavior. Moreover, turn detection demonstrated the impact of sensor placement very well.

Flexibility in sensor placement: Flexibility allows smartphone-based data collection or relaxes heavy sensor setups, which potentially increases the usability of motion tracking systems among recreational skiers due to ease of use while being reliable. Additionally, data collection in alpine skiing is challenging on its own, and employing such an approach will help fix sensor misplacement and misalignment.

Comparison to the state of the art: The aim of comparing our approach to other approaches in the literature [10,18,20,21] was not to reject their approach but to draw attention to the sensitivity of algorithms regarding sensor placement. As the results in Table 2 and Table 3 indicate, the other approaches fail in the case of sensor placement and orientation change, while our preprocessing method avoids such failures. In our study, we carefully located sensors on the skier’s body. However, if an error had naturally happened, it would have just served as another reason to highlight such an effect. We believe motion capturing in the future should be less sensitive to sensor misplacement since sensor misplacement and orientation change are not unlikely, considering the dynamic movements and environmental challenges inherent in alpine skiing.

Additionally, a closer look at Figure 5 reveals that turning patterns vary considerably according to the sensor placement. Consequently, even if the detected turning points or turn switches are accurate, the turning pattern cannot be extracted. Recording accurate skiing turning patterns, therefore, requires either a sensor carefully aligned with the CoM or multiple sensors. On the other hand, we proposed a method that does not force those limitations.

## 8. Conclusions

This study detailed the impact of sensor placement and orientation on the captured signals using IMUs during skiing, which is overlooked in the literature, and offered a motion analysis framework for alpine skiing to address the issues due to potential misplacement and orientation change. Using a quaternion-based complementary filter for sensor fusion and wavelet-driven side motion detection, we reconstructed consistent turning patterns on the side motion. Additionally, we achieved turn detection results with an overall error RMSE of 0.77 and MAE of 0.5 turns across all the sensors, subjects, and skiing techniques.

The novelty of our approach lies in its ability to operate with only a single sensor without requiring precise mounting or multiple sensors, marking an advance over existing algorithms that assume inflexible, carefully placed sensors (aligned with the CoM). This investigation attempts to open a new door in flexible sensor attachment to ease long-term data recording, especially using smartphone IMU sensors without losing turning patterns. Using the proposed approach, we can estimate the trajectory of the CoM while performing skiing activities independent of sensor location and orientation. As future work, we encourage researchers to adopt and adapt our approach in varied skiing contexts or in combination with other sensors, thereby extending its validation and fostering accessible, large-scale, and long-term performance monitoring.

## Figures and Tables

**Table 1:** Data collection. The experiment took place in varying conditions, such as in varying skiing locations, slopes, and seasons. * the entire recording time in minutes including skiing. ^+^ number of performed turns.

#	Location	When	Glacier	Sensor	Subjects	Duration (m) *	Turns ^+^
1	Hintertux, Tyrol	June 2019	Yes	Galaxy S9, Xsens	4	58	261
2	Dachstein, Upper Austria	November 2019	Yes	Galaxy S9, Xsens	2	48	129

**Table 2:** Turn detection results overall. Extracted turns from the side motion exhibit a consistent value with −1 to 2 error, while extracting turns from the other sensors is not reliable. However, the gyrX should be preferred over the other raw signals. * turn detection rate (std).

Input	RMSE	MAE	*TDR_std* *
accY	4.56	2.67	0.39
gyrX	2.67	1.15	0.22
Roll angle	6.14	3.69	0.37
Baseline	5.90	4.70	0.50
Side motion	0.77	0.50	0.09
Ground truth	0.00	0.00	0.00

**Table 3:** Turn detection error per sensor. Values for the ground truth and baseline stay the same as in Table 2. * turn detection rate (std).

Sensor Location	accY			gyrX			Roll Angle			Side Motion		
	RMSE	MAE	*TR_std* *	RMSE	MAE	*TR_std* *	RMSE	MAE	*TR_std* *	RMSE	MAE	*TR_std* *
Chest	0.00	0.00	0.00	1.13	0.64	0.10	3.77	1.27	0.16	0.77	0.50	0.09
Pelvis	0.00	0.00	0.00	0.66	0.44	0.08	6.32	2.88	0.24	0.71	0.38	0.08
Pelvis left—smartphone	5.82	3.27	0.35	1.00	0.64	0.13	6.74	4.27	0.35	0.85	0.64	0.10
Pelvis right—smartphone	4.92	3.00	0.39	1.64	1.05	0.21	5.40	2.64	0.29	0.85	0.55	0.09
Shoulder left	2.56	1.36	0.19	5.58	3.32	0.49	7.30	4.45	0.43	0.71	0.41	0.08
Shoulder right	4.88	2.64	0.30	5.45	3.23	0.42	5.53	3.59	0.38	0.77	0.50	0.09
Upper arm left	3.10	2.00	0.29	1.89	1.14	0.15	5.38	3.09	0.30	0.74	0.45	0.08
Upper arm right	2.47	1.55	0.21	4.45	1.77	0.23	7.38	4.95	0.31	0.83	0.50	0.09
Front arm left	5.20	3.68	0.37	1.02	0.68	0.13	8.31	6.05	0.33	0.71	0.41	0.09
Front arm right	7.02	5.41	0.53	0.83	0.59	0.11	7.26	5.05	0.36	0.77	0.50	0.10
Thigh left	4.68	3.14	0.41	2.24	1.18	0.15	5.91	3.82	0.37	0.77	0.50	0.10
Thigh right	1.33	0.77	0.23	0.85	0.64	0.10	6.75	3.77	0.36	0.80	0.55	0.09
Leg left	6.41	5.09	0.49	0.60	0.36	0.08	8.63	6.64	0.57	0.80	0.55	0.10
Leg right	6.47	4.90	0.53	0.45	0.20	0.05	7.91	6.05	0.52	0.71	0.50	0.07

## Data Availability

The data presented in this study are not publicly available, as informed consent from all but one subject was limited solely to the publication of study results. Anonymized data from the consenting participant may be made available from the corresponding author upon reasonable request.

## References

[1] Turnbull J., Kilding A., Keogh J. (2009). Physiology of alpine skiing. Scand. J. Med. Sci. Sport..

[2] Stead J., Poolton J., Alder D. (2022). Performance slumps in sport: A systematic review. Psychol. Sport Exerc..

[3] Hermann A., Senner V. (2021). Knee injury prevention in alpine skiing. A technological paradigm shift towards a mechatronic ski binding. J. Sci. Med. Sport.

[4] Zwölfer M., Heinrich D., Schindelwig K., Wandt B., Rhodin H., Spörri J., Nachbauer W. (2023). Deep learning-based 2D keypoint detection in alpine ski racing–A performance analysis of state-of-the-art algorithms applied to regular skiing and injury situations. JSAMS Plus.

[5] Supej M. (2010). 3D measurements of alpine skiing with an inertial sensor motion capture suit and GNSS RTK system. J. Sport. Sci..

[6] Yoshioka S., Fujita Z., Hay D.C., Ishige Y. (2018). Pose tracking with rate gyroscopes in alpine skiing. Sport. Eng..

[7] Qi J., Li D., Zhang C., Wang Y. (2022). Alpine skiing tracking method based on deep learning and correlation filter. IEEE Access.

[8] Dunnhofer M., Micheloni C. (2024). Visual tracking in camera-switching outdoor sport videos: Benchmark and baselines for skiing. Comput. Vis. Image Underst..

[9] Supej M., Holmberg H.C. (2021). Monitoring the performance of alpine skiers with inertial motion units: Practical and methodological considerations. J. Sci. Sport Exerc..

[10] Martínez A., Brunauer R., Venek V., Snyder C., Jahnel R., Buchecker M., Thorwartl C., Stöggl T. (2019). Development and validation of a gyroscope-based turn detection algorithm for alpine skiing in the field. Front. Sport. Act. Living.

[11] Azadi B., Haslgrübler M., Anzengruber-Tanase B., Sopidis G., Ferscha A. (2024). Robust Feature Representation Using Multi-Task Learning for Human Activity Recognition. Sensors.

[12] Federolf P.A. (2012). Quantifying instantaneous performance in alpine ski racing. J. Sport. Sci..

[13] Hébert-Losier K., Supej M., Holmberg H.C. (2014). Biomechanical factors influencing the performance of elite alpine ski racers. Sport. Med..

[14] Ruiz-García I., Navarro-Marchal I., Ocaña-Wilhelmi J., Palma A.J., Gómez-López P.J., Carvajal M.A. (2021). Development and evaluation of a low-drift inertial sensor-based system for analysis of alpine skiing performance. Sensors.

[15] Pérez-Chirinos Buxadé C., Padullés Riu J.M., Gavaldà Castet D., Trabucchi M., Fernández-Valdés B., Tuyà Viñas S., Moras Feliu G. (2022). Influence of Turn Cycle Structure on Performance of Elite Alpine Skiers Assessed through an IMU in Different Slalom Course Settings. Sensors.

[16] Vaverka F., Vodickova S. (2010). Laterality of the lower limbs and carving turns. Biol. Sport.

[17] Bon I., Očić M., Cigrovski V., Rupčić T., Knjaz D. (2021). What are kinematic and kinetic differences between short and parallel turn in Alpine skiing?. Int. J. Environ. Res. Public Health.

[18] Yu G., Jang Y.J., Kim J., Kim J.H., Kim H.Y., Kim K., Panday S.B. (2016). Potential of IMU sensors in performance analysis of professional alpine skiers. Sensors.

[19] Martínez A., Snyder C., Moore S.R., Stöggl T. (2021). A comprehensive comparison and validation of published methods to detect turn switch during alpine skiing. Sensors.

[20] Russo C., Puppo E., Roati S., Somà A. (2022). Proposal of an alpine skiing kinematic analysis with the aid of miniaturized monitoring sensors, a pilot study. Sensors.

[21] Debertin D., Wachholz F., Mikut R., Federolf P. (2022). Quantitative downhill skiing technique analysis according to ski instruction curricula: A proof-of-concept study applying principal component analysis on wearable sensor data. Front. Bioeng. Biotechnol..

[22] Klous M., Müller E., Schwameder H. (2010). Collecting kinematic data on a ski/snowboard track with panning, tilting, and zooming cameras: Is there sufficient accuracy for a biomechanical analysis?. J. Sport. Sci..

[23] Vaverka F., Vodickova S., Elfmark M. (2012). Kinetic analysis of ski turns based on measured ground reaction forces. J. Appl. Biomech..

[24] Martínez A., Nakazato K., Scheiber P., Snyder C., Stöggl T. (2020). Comparison of the turn switch time points measured by portable force platforms and pressure insoles. Front. Sport. Act. Living.

[25] Brodie M., Walmsley A., Page W. (2008). Fusion motion capture: A prototype system using inertial measurement units and GPS for the biomechanical analysis of ski racing. Sport. Technol..

[26] Supej M., Spörri J., Holmberg H.C. (2020). Methodological and practical considerations associated with assessment of alpine skiing performance using global navigation satellite systems. Front. Sport. Act. Living.

[27] Matsumura S., Ohta K., Yamamoto S.i., Koike Y., Kimura T. (2021). Comfortable and convenient turning skill assessment for alpine skiers using imu and plantar pressure distribution sensors. Sensors.

[28] Wang Y., Shao T., Jiang P., Shan G., Wang L., Li G. A Pilot Study on a Multimodal Wearable System by Applying a Two-Chain Biomechanical Model in the Alpine Ski Slalom. Proceedings of the 2021 IEEE International Conference on Real-time Computing and Robotics (RCAR).

[29] Khaked A.A., Oishi N., Roggen D., Lago P. Investigating the Effect of Orientation Variability in Deep Learning-based Human Activity Recognition. Proceedings of the Adjunct Proceedings of the 2023 ACM International Joint Conference on Pervasive and Ubiquitous Computing & the 2023 ACM International Symposium on Wearable Computing.

[30] Roggen D., Förster K., Calatroni A., Bulling A., Tröster G. On the issue of variability in labels and sensor configurations in activity recognition systems. Proceedings of the Best Practice in Activity Recognition Workshop at the 8th International Conference on Pervasive Computing.

[31] Zhong Y., Deng Y. Sensor orientation invariant mobile gait biometrics. Proceedings of the IEEE international joint conference on biometrics.

[32] STMicroelectronics (2017). LSM6DSL iNEMO Inertial Module: Always-On 3D Accelerometer and 3D Gyrosc.

[33] AKM (2015). AK09916 3-axis Electronic Compass. https://www.alldatasheet.com/html-pdf/917549/AKM/AK09916/54/1/AK09916.html.

[34] Paulich M., Schepers M., Rudigkeit N., Bellusci G. (2018). Xsens MTw Awinda: Miniature Wireless Inertial-Magnetic Motion Tracker for Highly Accurate 3D Kinematic Applications.

[35] Österreichische Skischule D. (2021). Snowsport Austria.

[36] Yan Z., Subbaraju V., Chakraborty D., Misra A., Aberer K. Energy-efficient continuous activity recognition on mobile phones: An activity-adaptive approach. Proceedings of the 2012 16th International Symposium on Wearable Computers.

[37] LaValle S.M., Yershova A., Katsev M., Antonov M. Head tracking for the Oculus Rift. Proceedings of the 2014 IEEE International Conference on Robotics and Automation (ICRA).

[38] LaValle S.M. (2023). Virtual Reality.

[39] Nazarahari M., Rouhani H. (2021). Sensor fusion algorithms for orientation tracking via magnetic and inertial measurement units: An experimental comparison survey. Inf. Fusion.

[40] Azadi B., Haslgrübler M., Anzengruber-Tanase B., Grünberger S., Ferscha A. (2022). Alpine skiing activity recognition using smartphone’s IMUs. Sensors.

[41] Torrence C., Compo G.P. (1998). A practical guide to wavelet analysis. Bull. Am. Meteorol. Soc..

[42] Cazelles B., Chavez M., Berteaux D., Ménard F., Vik J.O., Jenouvrier S., Stenseth N.C. (2008). Wavelet analysis of ecological time series. Oecologia.

[43] Martínez A., Jahnel R., Buchecker M., Snyder C., Brunauer R., Stöggl T. (2019). Development of an automatic alpine skiing turn detection algorithm based on a simple sensor setup. Sensors.

[44] Young A.D. Comparison of orientation filter algorithms for realtime wireless inertial posture tracking. Proceedings of the 2009 Sixth International Workshop on Wearable and Implantable Body Sensor Networks.

[45] Nazarahari M., Rouhani H. (2021). 40 years of sensor fusion for orientation tracking via magnetic and inertial measurement units: Methods, lessons learned, and future challenges. Inf. Fusion.

[46] Tuck K. (2007). Tilt Sensing Using Linear Accelerometers. Freescale Semiconductor Application Note AN3107. https://www.nxp.com/docs/en/application-note/AN3461.pdf.

